# Characterization of Three New Glutaredoxin Genes in the Arbuscular Mycorrhizal Fungus *Rhizophagus irregularis*: Putative Role of RiGRX4 and RiGRX5 in Iron Homeostasis

**DOI:** 10.1371/journal.pone.0149606

**Published:** 2016-02-22

**Authors:** Elisabeth Tamayo, Karim Benabdellah, Nuria Ferrol

**Affiliations:** 1 Departamento de Microbiología del Suelo y Sistemas Simbióticos, Estación Experimental del Zaidín, Consejo Superior de Investigaciones Científicas, Granada, Spain; 2 Genomic Medicine Department, GENYO, Centre for Genomics and Oncological Research, Pfizer-University of Granada-Andalusian Regional Government, Parque Tecnológico Ciencias de la Salud, Granada, Spain; IPK, GERMANY

## Abstract

Glutaredoxins (GRXs) are small ubiquitous oxidoreductases involved in the regulation of the redox state in living cells. In an attempt to identify the full complement of GRXs in the arbuscular mycorrhizal (AM) fungus *Rhizophagus irregularis*, three additional GRX homologs, besides the formerly characterized GintGRX1 (renamed here as RiGRX1), were identified. The three new GRXs (RiGRX4, RiGRX5 and RiGRX6) contain the CXXS domain of monothiol GRXs, but whereas RiGRX4 and RiGRX5 belong to class II GRXs, RiGRX6 belongs to class I together with RiGRX1. By using a yeast expression system, we observed that the newly identified homologs partially reverted sensitivity of the GRX deletion yeast strains to external oxidants. Furthermore, our results indicated that RiGRX4 and RiGRX5 play a role in iron homeostasis in yeast. Gene expression analyses revealed that *RiGRX1* and *RiGRX6* were more highly expressed in the intraradical (IRM) than in the extraradical mycelium (ERM). Exposure of the ERM to hydrogen peroxide induced up-regulation of *RiGRX1*, *RiGRX4* and *RiGRX5* gene expression. *RiGRX4* expression was also up-regulated in the ERM when the fungus was grown in media supplemented with a high iron concentration. These data indicate the two monothiol class II GRXs, RiGRX4 and RiGRX5, might be involved in oxidative stress protection and in the regulation of fungal iron homeostasis. Increased expression of *RiGRX1* and *RiGRX6* in the IRM suggests that these GRXs should play a key role in oxidative stress protection of *R*. *irregularis* during its *in planta* phase.

## Introduction

Arbuscular mycorrhizal fungi (AM fungi) are soil microorganisms belonging to the Glomeromycota phylum that establish mutualistic symbioses, called arbuscular mycorrhizas (AM), with the roots of the majority of higher plants. In this symbiosis, the fungus provides the plant with mineral nutrients of low mobility in the soil and in return the fungus receives carbon supplies from the plant [1]. The AM symbiosis benefits plants not only promoting growth but also enhancing plant tolerance to biotic and abiotic stresses [2]. In the course of the symbiosis, roots are colonized by fungal hyphae that ultimately form intracellular tree-like structures termed arbuscules in the inner-cortical cells, facilitating nutrient exchange between the two partners. The establishment of such an intimate interaction, allowing the fungus to grow intracellularly in the host cells, requires its recognition as a symbiotic partner and a tight regulation of processes leading to the accommodation of the beneficial fungus. At the molecular level, this process is only partly understood, and the precise function of most plant genes known to be regulated during fungal colonization remains elusive [3–6]. On the fungal side, knowledge of the mechanisms underlying adaptation to the symbiotic mode is even more limited. This is mainly due to the fact that research on AM fungi is hampered by their obligate biotrophic life style and that they are so far not amenable to genetic manipulation. However, the recently published genome and genome-wide transcriptomic data of an AM fungus open new opportunities [7–9].

The AM association shares several common features with those of plant-fungal pathogens, including local and transient production of reactive oxygen species (ROS) [10,11], induction of plant defence genes [12] and the use of effector proteins to counteract plant defence responses [13]. In AM roots, accumulation of hydrogen peroxide (H_2_O_2_) has been observed around the hyphal tips attempting to penetrate a host cell and in cells containing arbuscules, while no H_2_O_2_ accumulation was observed in hyphal tips growing along the middle lamella, appressoria or vesicles [14]. Recent studies provided evidence that ROS concentrations tightly control the outcome of the symbiosis. Silencing of genes involved in ROS production, such as ROP9, a small GTPase from *Medicago truncatula* [15] or the *Phaseolus vulgaris* NADPH oxidase RbohB [16], induced early hyphal colonization and enhanced root length colonization, while an enhanced accumulation of ROS in over-expressing RbohB *P*. *vulgaris* roots impaired AM fungal colonization [17]. However, in *M*. *truncatula* it has been shown that silencing of RbohE, a NADPH oxidase isoform that is expressed in arbuscule-containing cells, induced an altered colonization pattern in the root cortex with fewer arbuscules and multiple penetration attempts [18]. Fungal suppression of ROS-mediated defence by the secretion of ROS-scavenging enzymes, such as the superoxide dismutase [19,20], or the production of antioxidant compounds, such as vitamin B6 [21], has been proposed to be necessary for successful colonization of the host plant by AM fungi [19]. In addition to the mediation of plant–fungal interactions by host-derived ROS, the endogenous production of ROS by AM fungi should be important for their normal development and adaptation to environmental stresses. In spite of the central role that gluthatione (GSH) plays as a cellular redox buffer, only one enzyme related with this metabolite, a dithiol glutaredoxin (GRX), has been characterized in an AM fungus [22].

Glutaredoxins (GRXs) are small ubiquitous oxidoreductases that mediate reversible reduction of the disulphide bonds formed between cysteine residues of proteins and glutathione via a dithiol or monothiol mechanism. GRXs generally contain a conserved CXXC/S or CGFS active-site motif, which is involved in the reduction reaction, and the TVP and GG motifs involved in glutathione binding [23,24]. GRXs were initially classified into two groups, dithiol and monothiol, according to their active site sequence, having two or one cysteine residues in it, respectively. Nevertheless, due to the discovery of an increasing number of GRX sequences, Couturier et al. [25] and afterwards Ströher and Millar [26] proposed a new classification based on sequence structure, in which non-plant GRXs were categorized into classes I and II. Class I includes GRXs containing dithiol or monothiol active-site motifs (CXXC/S), whereas class II contains all GRXs with a CGFS motif in their active site [27]. GRXs have been shown to be involved in the maintenance and regulation of the cellular redox state and in redox-dependent signalling pathways [28]. Due to the general importance of these processes, these enzymes are involved in diverse cellular processes and play an important role in defence against oxidative stress [23]. The majority of Class II GRXs characterized to date have been shown to be required for the formation of iron-sulfur clusters [29] or as chaperons for the transfer and delivery of iron-sulfur clusters to acceptors apoproteins [30], playing a role in iron metabolism.

Glutaredoxins have been extensively studied in *Saccharomyces cerevisiae* [31,32] and *Schizosaccharomyces pombe* [33] and *Sinorhizobium meliloti* [34]. In the nitrogen-fixing symbiosis established between *S*. *meliloti* and *Medicago* plants, two *S*. *meliloti* GRX proteins have been shown to be essential for optimal development and functioning of the nitrogen-fixing symbiosis, playing independent roles in deglutathionylation reactions and in the regulation of iron metabolism. In the AM fungus *R*. *irregularis* only a dithiol GRX, GintGRX1, displaying oxidoreductase, peroxidase and glutathione S-transferase activities has been reported [22]. With the aim of getting further insights into the roles of GRXs in AM fungi, a genome-wide approach has been used to identify and characterize the complete set of genes encoding GRXs in the AM fungus *R*. *irregularis*. Our data indicate that the *R*. *irregularis* GRX gene family is composed of four members. Characterization of the identified genes indicates that the different *R*. *irregularis* isoforms play diverse roles in the fungus and that the two monothiol class II GRXs, RiGRX4 and RiGRX5, might be involved in oxidative stress protection and in the regulation of fungal iron homeostasis.

## Materials and Methods

### Biological materials and growth conditions

*Rhizophagus irregularis* monoxenic cultures were established as described by St-Arnaud et al. [35], with some modifications. Briefly, clone DC2 of carrot (*Daucus carota* L.) Ri-T DNA transformed roots were cultured with the AM fungus *R*. *irregularis* Schenck and Smith DAOM 197198 in two-compartment Petri dishes. Cultures were initiated in one compartment of each plate containing M medium [36] by placing several non-mycorrhizal carrot root segments and a piece of fungal inoculum containing extraradical mycelium (ERM), fragments of mycorrhizal roots and spores. Fungal hyphae and roots were allowed to grow over to the other compartment containing the same M medium. Plates were incubated in the dark at 24°C for 7–8 weeks until the second compartment was profusely colonized by the fungus and the roots. Then, the older compartment was removed and refilled with liquid M medium without sucrose (M-C medium) containing different iron concentrations: 45 μM (control), 4.5 mM or 45 mM EDTA iron(III) sodium salt. Fungal hyphae, but not roots, were allowed to grow over to this compartment (hyphal compartment). Plates were incubated in the dark at 24°C for 2–3 additional weeks. For the oxidative stress treatments, the fungus grown in control liquid M-C medium was exposed for 0.5, 1 and 2 h to 0.1 or 1 mM H_2_O_2_. This was done by replacing the liquid medium of the hyphal compartment of a control plate by 15 ml of a freshly prepared liquid M-C medium supplemented with H_2_O_2_. The control plates received 15 ml of liquid M-C medium.

ERM from the different hyphal compartments was directly recovered under sterile conditions by using a pair of tweezers, washed with sterile water and dried on filter paper. The mycelium was immediately frozen in liquid nitrogen and stored at -80°C until used.

To analyze intraradical gene expression, hyphae growing in the hyphal compartment were used as a source of mycorrhizal inoculum. Carrot roots were placed on top of a densely colonized hyphal compartment and collected 15 days later. Extraradical hyphae attached to the roots were removed with forceps under a binocular microscope. Roots were then frozen in liquid N and stored at -80°C until used. Furthermore, rice roots (*Oryza sativa* L. cv. Nipponbare) colonized by *R*. *irregularis* were also used. Rice plants were grown as described in Pérez-Tienda et al. [37]. Briefly, rice seedlings from seeds germinated in autoclaved vermiculite were transplanted into pots containing a sterile mixture of soil:sand:vermiculite (1:2:6, v:v:v), and plants were grown in a growth chamber with 23/18°C day/night temperature, 60% relative humidity and 16/8 h light/dark photoperiod. Mycorrhizal inoculation was performed using a sepiolite-vermiculite-based inoculum of *R*. *irregularis* (10%, v/v), containing spores, hyphae and fragments of AM roots. Control plants (non-mycorrhizal) received the same proportion of the inoculum substrate and an aliquot of a filtrate (<20 μm) of the AM inoculum to provide the microbial populations accompanying *R*. *irregularis* but free from AM propagules. Roots were harvested 8 weeks after inoculation, gently washed under tap water to try to eliminate most of the attached fungal hyphae and spores, frozen in liquid nitrogen and stored at -80°C until used. Mycorrhizal root colonization was estimated after trypan blue staining according to the grid-line intersect method using a stereomicroscope [38].

The *S*. *cerevisiae* strains used in this study were all isogenic derivatives of CML128, W303-1A and YPH449 wild type strains (Table 1). The *Δgrx3Δgrx4* mutant MML449, the *Δgrx5* mutant MML100 and their respective parental strains CML128 and W303-1A were kindly provided by Dr. Enrique Herrero (Departament de Ciències Mèdiques Bàsiques, Facultat de Medicina, Universitat de Lleida, Lleida, Spain). The *Δgrx6Δgrx7* yeast strain and its parental YPH449 were kindly provided by Dr. Johannes M. Herrmann (Department of Cell Biology, University of Kaiserslautern, Kaiserslautern, Germany). Strains were grown on YPD or minimal synthetic dextrose (SD) medium, supplemented with appropriate amino acids.

**Table 1:** *S*. *cerevisiae* strains used in this work.

Strain	Relevant genotype	Reference
W303-1A	*MATa ura3–1 ade2–1 leu2–3*,*112 trp1–1 his3–11*,*15*	[39]
CML128	*MATa leu2-3*,*112 ura3-52 trp1 his4 can1*^*r*^	[40]
YPH449	*MATa ura3-52 lys2-801 ade2-101 trp1-Δ63 his3-Δ200 leu2-Δ1*	[41]
MML100	*MATa grx5*::*kanMX4* as W303-1A	[42]
MML449	*MATa grx3*::*natMX4 grx4*::*kanMX4* as CML128	[43]
*Δgrx6Δgrx7*	*MATa grx6*::*HIS3 grx7*::*kan* as YPH449	[44]
MML100 pFL61	*Δgrx5* transformed with the empty vector	This work
MML100 pFL61Sc*Grx5*	*Δgrx5* transformed with the construct pFL61*ScGrx5*	This work
MML100 pFL61*RiGRX5*	*Δgrx5* transformed with the construct pFL61*RiGRX5*	This work
MML100 pFL61*RiGRX4*	*Δgrx5* transformed with the construct pFL61*RiGRX4*	This work
MML100 pFL61*RiGRX6*	*Δgrx5* transformed with the construct pFL61*RiGRX6*	This work
MML449 pFL61	*Δgrx3Δgrx4* transformed with the empty vector	This work
MML449 pFL61*ScGrx3*	*Δgrx3Δgrx4* transformed with the construct pFL61*ScGrx3*	This work
MML449 pFL61*ScGrx4*	*Δgrx3Δgrx4* transformed with the construct pFL61*ScGrx4*	This work
MML449 pFL61*RiGRX4*	*Δgrx3Δgrx4* transformed with the construct pFL61*RiGRX4*	This work
MML449 pFL61*RiGRX5*	*Δgrx3Δgrx4* transformed with the construct pFL61*RiGRX5*	This work
MML449 pFL61*RiGRX6*	*Δgrx3Δgrx4* transformed with the construct pFL61*RiGRX6*	This work
MML449 pBL106	*Δgrx3Δgrx4* transformed with pBL106	This work
MML449 pBL106*RiGRX4*	*Δgrx3Δgrx4* transformed with the construct pBL106*RiGRX4*	This work
MML449 pBL106*RiGRX5*	*Δgrx3Δgrx4* transformed with the construct pBL106*RiGRX5*	This work
*Δgrx6Δgrx7* pFL61	*Δgrx6Δgrx7* transformed with the empty vector	This work
*Δgrx6Δgrx7* pMM822	*Δgrx6Δgrx7* transformed with a plasmid containing the *ScGrx6* open reading frame [45]	This work
*Δgrx6Δgrx7* pFL61*RiGRX6*	*Δgrx6Δgrx7* transformed with the construct pFL61*RiGRX6*	This work
*Δgrx6Δgrx7* pBL106	*Δgrx6Δgrx7* transformed with pBL106	This work
*Δgrx6Δgrx7* pBL106*RiGRX6*	*Δgrx6Δgrx7* transformed with the construct pBL106*RiGRX6*	This work

### Nucleic acids extraction and cDNA synthesis

*Rhizophagus irregularis* genomic DNA was extracted from ERM developed in the hyphal compartment of control plates using the DNeasy Plant Mini Kit (Qiagen), according to the manufacturer’s instructions.

Total plant RNA was isolated from rice roots using the phenol/SDS method followed by LiCl precipitation as described by García-Rodríguez et al. [46]. Total fungal RNA from ERM from the different treatments of *R*. *irregularis* and mycorrhizal carrot roots, was extracted using the RNeasy Plant Mini Kit (QIAGEN, Maryland), following manufacturer′s instructions. DNAse treatment was performed using the RNA-free DNase set (QIAGEN, Maryland) following the manufacturer’s instructions. cDNAs were obtained from 1 μg of total DNase-treated RNA in a 20 μl reaction containing 200 units of Super-Script III Reverse Transcriptase (Invitrogen) and 50 pmol oligo (dT)_20_ (Invitrogen), according to the manufacturer’s instructions.

### Identification of *GRX* genes in *R*. *irregularis* and sequences analyses

Amino acid sequences of *S*. *cerevisiae* GRXs were retrieved from the freely accessible *S*. *cerevisiae* genome database (http://www.yeastgenome.org/) and used to search for orthologous sequences in the filtered model dataset of *R*. *irregularis* on the JGI website (http://genome.jgi.doe.gov/Gloin1/Gloin1.home.html; [7,8]) using Basic Local Alignment Search Tool (BLAST) algorithm [47] via a protein BLAST. A second search was performed via a keyword search directly.

Predictions of putative transmembrane domains were made using the TMHMM Server v.2.0 (http://www.cbs.dtu.dk/services/TMHMM/) and SMART software (http://smart.embl-heidelberg.de/). Predictions of subcellular localizations were made using the TargetP 1.1 Server (http://www.cbs.dtu.dk/services/TargetP/), PSORTII (http://psort.hgc.jp/form2.html) and WoLF PSORT (http://wolfpsort.org/).

Full-length amino acid sequences were aligned with the orthologous sequences of a number of fungi representatives of distinct taxonomic groups by ClustalW (Version 2.1 [48]; http://www.ebi.ac.uk/Tools/msa/clustalw2/). Alignments were imported into the Molecular Evolutionary Genetics Analysis (MEGA) package version 6 [49]. Phylogenetic analyses were conducted by the neighbour-joining (NJ) method, implemented in MEGA, with a pair-wise deletion of gaps and the Poisson model for distance calculation. Bootstrap analyses were carried out with 1000 replicates. The evolutionary tree was drawn to scale. Weblogo was used to generate the sequence logos of glutathione binding site and active site motifs (http://weblogo.berkeley.edu/) [34,50].

### Gene isolation

The full-length cDNAs of *RiGRX4*, *RiGRX5* and *RiGRX6* were obtained by PCR amplification of *R*. *irregularis* cDNA obtained from ERM growing in control plates, using the primer pairs Grx4.fF and Grx4.fR, Grx5.fF and Grx5.fR and Grx7.fF and Grx7.fR, respectively. Sequences of the primers used are listed in S1 Table. *RiGRX4* and *RiGRX5* PCR products were cloned in the pCR2.1 vector (Invitrogen, Carlsbad, CA, USA) and *RiGRX6* in the pGEM-T Easy vector (Promega, Madison, USA).

All plasmids were amplified by transformation of *E*. *coli* following standard procedures and purified by using the Qiagen Miniprep Kit (Qiagen, Maryland, USA). All sequences and constructs were checked by sequencing before further use. Nucleotide sequences were determined by Taq polymerase cycle sequencing by using an automated DNA sequencer (ABI Prism 3130xl Genetic Analyzer, Applied Biosystems, Carlsbad, USA).

### Heterologous expression and growth assays

For heterologous RiGRXs expression analyses, the full length cDNAs were cloned into the yeast expression vector pFL61 [51]. To obtain pFL61-*RiGRX4*, pFL61-*RiGRX5* and pFL61-*RiGRX6*, the respective full-length cDNAs were isolated from the pCR2.1 or pGEM-T easy vector by *Not*I digestion and ligated into the *Not*I-digested pFL61 vector. The full length cDNAs of *ScGrx3*, *ScGrx4* and *ScGrx5* were also cloned into pFL61 and used as positive controls in the complementation analyses of the *Δgrx3Δgrx4* and *Δgrx5* strains.

The *S*. *cerevisiae* mutant strains *Δgrx3Δgrx4*, *Δgrx5* and *Δgrx6Δgrx7* were transformed with the constructs pFL61-*RiGRX4*, pFL61-*RiGRX5*, pFL61-*RiGRX6*, the empty vector (negative control), or the corresponding positive controls using a lithium acetate-based method [52]. Yeast transformants were selected on SD medium by autotrophy to uracil.

For oxidant sensitivity determination, cells from exponentially growing cultures of the transformed yeast strains were 1:10 serial diluted and spotted onto SD plates containing or not the oxidizing agents. Lysine auxotrophy assessment of the transformed *Δgrx5* strains was assayed on SD medium with or without lysine.

To test sensitivity to CaCl_2_ of the transformed *Δgrx6Δgrx7* strains, cells grown in SD medium to exponential phase (OD_600_ = 0.6–0.8) were harvested, washed twice and resuspended in the same volume of a SD modified medium (0.2% YNB w/o amino acids and ammonium sulphate (Difco) with 76 mM NH_4_Cl_2_ as nitrogen source [53]) supplemented or not with 500 mM CaCl_2_. Growth of the treated and untreated cells was recorded (OD_600_) at 1 –h intervals until the stationary phase was reached. The ratio of growth values between treated and untreated cells after different periods of time was calculated and then made relative to the same ratio in the positive control (*Δgrx6Δgrx7* transformed with the *S*. *cerevisiae Grx6*).

### Measurement of iron concentration

The *Δgrx3Δgrx4* and *Δgrx5* yeast strains transformed with either the empty vector, pFL61-*RiGRX4*, pFL61-*RiGRX5*, pFL61-*RiGRX6* or their respective positive control construct were grown in 50 ml of SD media overnight at 30°C. Each yeast culture was washed twice, resuspended in YPD medium, diluted into 300 ml of YPD medium (OD_600_ 0.2–0.3) and grown until an OD_600_ of 1.0 was reached. Cells were collected by centrifugation, washed with 50 mM Tris pH 7.5, resuspended in 50 mM Tris pH 7.5 containing 0.15 M NaCl and disrupted with glass beads for 10 min at 4°C. Cell homogenates were centrifuged (7000 rpm, 4°C, 5 min) and the supernatants used as cell extracts.

The intracellular iron content was examined with a QuantiChrom^™^ iron assay kit (BioAssay Systems, Hayward, CA) following the manufacturer′s instructions.

### Enzyme assays

Cell extracts of the *Δgrx5* strain transformed with pFL61-*RiGRX4*, pFL61-*RiGRX5*, pFL61-*ScGrx5* or the empty vector were prepared in 0.1 M Tris pH 8.1, 2 mM EDTA and 1mM PMSF using glass beads to disrupt the cells.

Malate dehydrogenase activity was measured by following the consumption of NADH (ε = 6.22 mM^-1^ cm^-1^) spectrophotometrically at 340 nm [54]. Aconitase activity was assessed by measuring the absorption of converted NADPH (ε = 6.22 mM^-1^ cm^-1^) at 340 nm [55].

Protein content was determined by the Bio-Rad Protein Assay, using BSA as standard.

### Protein localization analyses

Localization of the *R*. *irregularis* GRX4, GRX5 and GRX6 proteins in *S*. *cerevisiae* was performed with C-terminal fusions of the respective genes to the enhanced green fluorescence protein (GFP) gene in the *Δgrx3Δgrx4* or *Δgrx6Δgrx7* strains. Gene-specific primer pairs containing *Sfi*IA (GGCCATTACGGCC) and *Sfi*IB (GGCCGAGGCGGCC) overhangs (S1 Table) were used to clone the three glutaredoxin cDNAs into the *Sfi*I sites of the plasmid pBL106, a pDR196sfi vector derivative carrying *GFP* [56]. The resulting plasmids pBL106-*RiGRX4* and pBL106-*RiGRX5* were used to transform the *Δgrx3Δgrx4* strain and pBL106-*RiGRX6* to transform the *Δgrx6Δgrx7* strain. Both strains were also transformed with the pBL106 empty vector. Cells were grown in CSM liquid culture to mid-logarithmic phase (OD_600_≈0.6–0.7), washed twice and resuspended in water for direct visualization. For labeling of yeast mitochondria, MitoTracker^®^ Red CM-H_2_XRos (Molecular Probes) was added to 1 ml of a mid-logarithmic phase suspension of the *RiGRX6*-*GFP*-expressing *Δgrx6Δgrx7* cells (final concentration: 200 nM). Labeling was performed for 1 hour at 30°C without subsequent washing of cells before visualization. The fluorescence signal was visualized with a Nikon Eclipse 50i fluorescent microscope. A 510–560 nm filter was used for MitoTracker^®^ Red CM-H_2_XRos fluorescence, and GFP fusion proteins were imaged using a 450–490 nm filter. Image sets were processed and overlapped using Adobe Photoshop^™^.

### Gene expression analyses

*RiGRX*s gene expression was studied by real-time RT-PCR using an iQ^™^5 Multicolor Real-Time PCR Detection System (Bio-Rad). Each 20 μl reaction contained 1 μl of a 1:10 dilution of the cDNA, 200 nM each primer, 10 μl of iQ^™^ SYBR Green Supermix 2x (Bio-Rad). The PCR program consisted in a 3 min incubation at 95°C, followed by 36 cycles of 30 s at 95°C, 30 s at 58°C and 30 s at 72°C, where the fluorescence signal was measured. The specificity of the PCR amplification procedure was checked with a heat-dissociation protocol (from 58 to 95°C) after the final cycle of the PCR. The primer sets used were GintGRXfw2 and GintGRXrev2 for *RiGRX1*; RiGrx4.qF and RiGrx4.qR for *RiGRX4*; RiGrx5.qF and RiGrx5.qR for *RiGRX5* and RiGrx7.qF and RiGrx7.qR for *RiGRX6* (S1 Table). The efficiency of the different primer sets was evaluated by performing real-time PCR on several dilutions of cDNA. Because RNA extracted from mycorrhizal roots contains plant and fungal RNAs, specificity of the primer pairs was also analyzed by PCR amplification of genomic DNA isolated from non-mycorrizal carrot roots and rice leaves and of cDNA from non-colonized carrot and rice roots. The results obtained for the different treatments were standardized to the elongation factor 1-alpha gene levels (GenBank Accession No. DQ282611), which were amplified with the primers GintEFfw and GintEFrev. RT-PCR determinations were performed on at three independent biological samples from three replicate experiments. Real-time PCR experiments were carried out three times for each biological sample, with the threshold cycle (Ct) determined in triplicate. The relative levels of transcription were calculated by using the 2^-ΔΔCT^ method [57], and the standard error was computed from the average of the ΔCT values for each biological sample.

### Statistical analyses

Statgraphics Centurion XVI software was used for the statistical analysis of the means and standard deviation determinations. ANOVA, followed by a Fisher’s LSD test (*p*<0.05) when possible, was used for the comparison of the treatments based on at least 3 biological replicates for each treatment (n ≥ 3).

## Results

### Identification of three new members of the GRX family in *R*. *irregularis*

A search for putative *GRX* genes in the *R*. *irregularis* genome led to the identification of five genes encoding proteins displaying significant sequence similarity to glutaredoxins, which were named according to their orthologous in *S*. *cerevisiae*: *RiGRX1* (GenBank Accession No. B7ZFT1; JGI ID 350295, formerly named GintGRX1 in Benabdellah *et al*. [22]), *RiGRX4* (GenBank Accession No. ESA11920; JGI ID 347595), *RiGRX5* (GenBank Accession No. ESA01501; JGI ID 337939), *RiGRX6* (GenBank Accession No. ESA04895; JGI ID 350273) and a hypothetical protein containing a GRX-like domain but whose overall sequence was closer to thioredoxins than to glutaredoxins (GenBank Accession No. ESA18464; JGI ID 344567).

The length of the nucleotide coding sequences of the four *RiGRX* genes ranged from 306 to 1002 bp. The coding exon sequences for the *RiGRX* genes were confirmed by cDNA sequencing. The number of introns in the individual *RiGRX*s, taken from the *R*. *irregularis* JGI database and confirmed by the comparison between the genomic sequences from JGI and the isolated coding sequences, varies from 2 to 7, and with the exception of the fourth intron of *RiGRX4* which contains a non-canonical GC/AG splicing sequence, all of them were flanked by the characteristic splicing sequences GT and AG at the 5’ and 3’ ends, respectively (S1 Fig).

**Fig 1:**
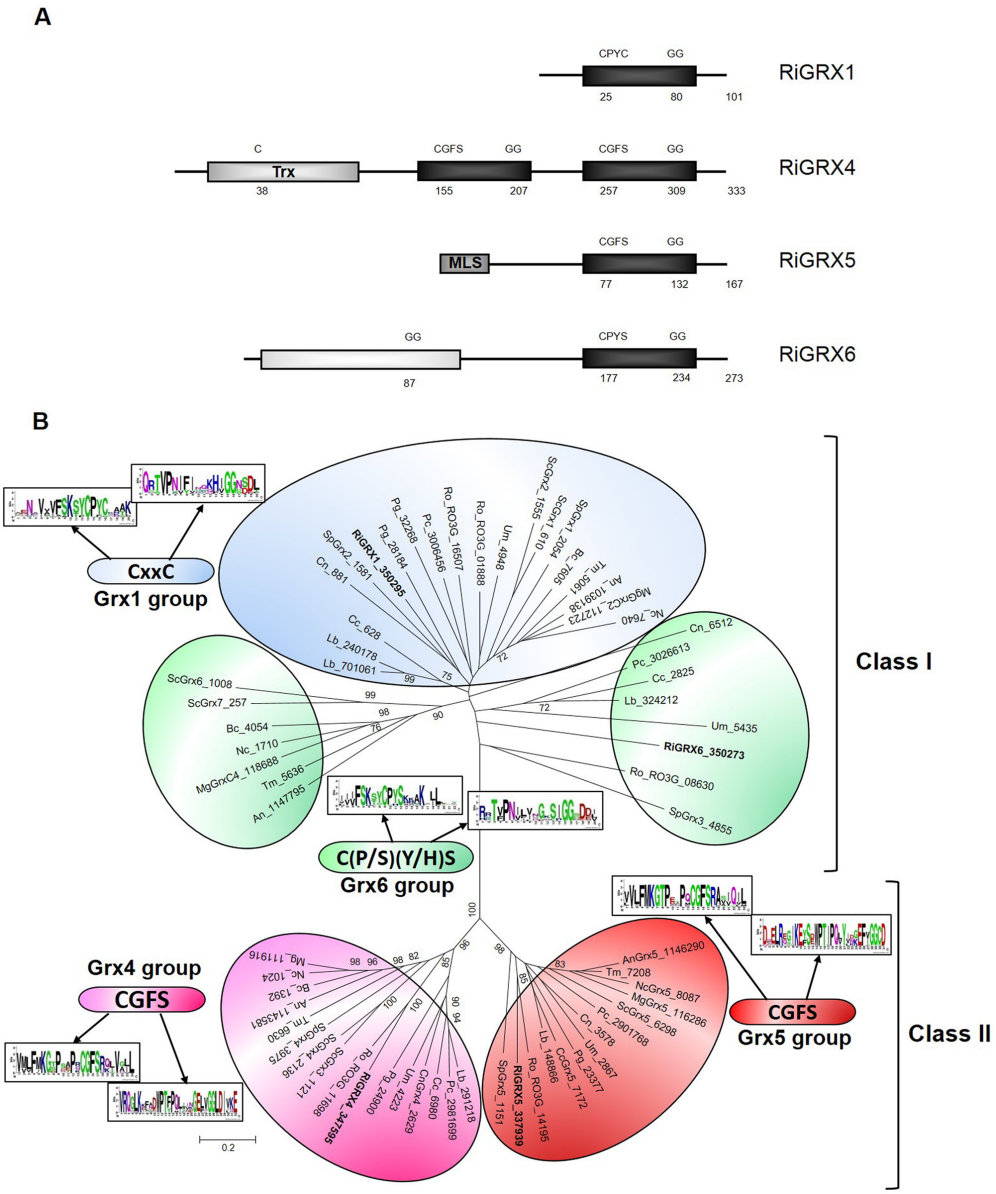
**A. Domain organization of the *R*. *irregularis* GRXs.** Glutaredoxin domains are represented by black boxes. The thioredoxin-like (Trx) domain of RiGRX4, the mitochondrial location signal (MLS) of RiGRX5 and the domain of unknown function of RiGRX6 (white box) are also indicated. Numbers correspond to the position of the first cysteine in the active site in the GRX domains, the first glycine of the glutathione binding domains and the total length of the proteins. The position of the cysteine in the Trx domain of RiGRX4 is also indicated. **B. Unrooted Nieghbor-Joining tree of the GRX family in fungi.** Organisms: An, *Aspergillus niger*; Bc, *Botrytis cinerea*; Cc, *Coprinopsis cinerea*; Cn, *Cryptococcus neoformans*; Lb, *Laccaria bicolor;* Mg, *Magnaporthe grisea*; Nc, *Neurospora crassa*; Pc, *Phanerochaete chrysosporium*; Pg, *Puccinia graminis;* Ri, *Rhizophagus irregularis*; Ro, *Rhizopus oryzae*; Sc, *Saccharomyces cerevisiae*; Sp, *Schizosaccharomyces pombe*; Tm, *Tuber melanosporum*; Um, *Ustilago maydis*. *R*. *irregularis* GRXs are emphasized in bold. Protein JGI identification numbers are indicated. *R*. *oryzae* sequences were retrieved from the Broad Institute databases (http://www.broad.mit.edu/annotation/). Bootstrap values above 70 and supporting a node are indicated.

RiGRX1 contains the CPYC active site typical of classical dithiol GRXs and, as previously reported by Benabdellah et al. [22] exhibits glutathione-disulfide oxidoreductase activity *in vitro*, while RiGRX4 and RiGRX5 possess the CGFS typical domain of classical monothiol GRXs (Fig 1A). RiGRX4 is a protein of 333 amino acids that consists of one thioredoxin domain followed by two monothiol GRX domains. RiGRX4 displays higher similarity to its vertebrate homologues, also containing two GRX domains (50% identity), than to yeast Grx3 (35% identity) and Grx4 (37% identity), with one GRX domain each. RiGRX5 (167 amino acids) has a single monothiol GRX domain and is more closely related to its *Cryptococcus neoformans* homolog (49% identity) than to *S*. *cerevisiae* Grx5 (37% identity). RiGRX4 is predicted to be cytosolic and RiGRX5 mitochondrial. *RiGRX6* encodes a protein of 273 amino acids that contains a CPYS motif at the active site, an N terminal domain of unknown function composed of 150 amino acids and a putative transmembrane domain close to the N-terminus. RiGRX6 shows the highest homology to its *Laccaria bicolor* homolog (44% identity) and is predicted to be in the secretory pathway. The *S*. *cerevisiae* homologs are ScGrx6 and ScGrx7, which are integral components of endoplasmic reticulum/Golgi membranes sharing sequence homology with dithiol GRXs but containing a single Cys residue at the active site.

A phylogenetic analysis of fungal GRXs revealed that RiGRX1 and RiGRX6 belong to class I GRXs and RiGRX4 and RiGRX5 to class II [25]. RiGRX1 clustered with the classical dithiol isoforms (GRX1 group) while RiGRX6 clustered with the Grx6 homologs. The GRX6 group is formed by proteins with C(P/S)(Y/H)S active-site sequences and splits in two subgroups, one clustering all the Ascomycota sequences except the *S*. *pombe* homolog (SpGrx3p) and the other clade grouping the Basidiomycota, Mucoromycotina and Glomeromycota homologs (Fig 1B).

### *RiGRXs* are differentially expressed in the intraradical and extraradical mycelium

As a first step to get some insigths into the role of the different *RiGRXs* in *R*. *irregularis*, quantitative gene expression analysis was performed by real-time RT-PCR on ERM collected from the hyphal compartment of *R*. *irregularis* monoxenic cultures, on carrot mycorrhizal roots developed in a densely colonized hyphal compartment of the split Petri dishes lacking ERM and on mycorrhizal rice roots developed in pot cultures and devoid of external hyphae. Mycorrhizal colonization level of the carrot and rice roots was 10 and 25%, respectively.

**Fig 2:**
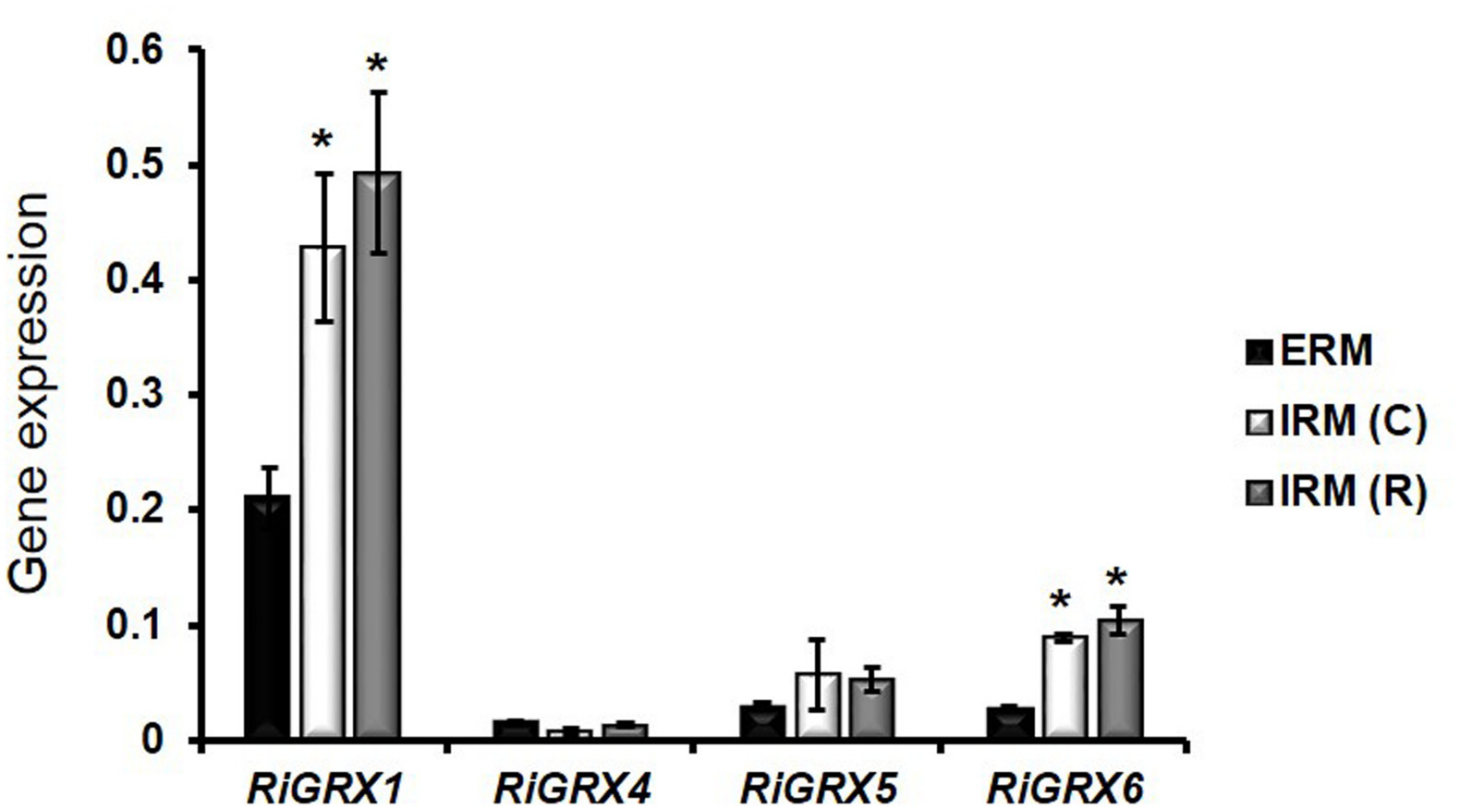
Relative expression of the *RiGRX* genes in extraradical mycelia (ERM) and intraradical (IRM) mycelia of *R*. *irregularis*. *RiGRX* gene expression was assessed in ERM developed in monoxenic cultures (ERM), *R*. *irregularis*-colonized carrot roots grown in monoxenic cultures and lacking ERM (IRM(C)) and *R*. *irregularis*-colonized rice roots grown in pot cultures and devoid of ERM (IRM(R)). Samples were normalized using the housekeeping gene *RiTEF*. Relative expression levels were calculated by the 2^-ΔCT^ method. Data are means +/- standard error. Asterisks show statistically significant differences (p<0.05) relative to the ERM, according to the Fisher’s LSD test.

*RiGRX1*, encoding a dithiol GRX, was the GRX isoform more highly expressed both in the ERM and in the mycorrhizal roots (IRM). Transcript levels of *RiGRX1* were 2- and 2.3-fold higher in the IRM of monoxenically grown carrot roots and of the rice mycorrhizal roots than in the ERM, respectively. Expression levels of *RiGRX6* were also higher in the IRM of carrot (3.2-fold increase) and rice (3.75-fold increase) mycorrhizal roots than in the ERM. No significant differences were observed between the expression levels of *RiGRX4* and *RiGRX5* in both fungal structures (Fig 2).

### RiGRXs suppress sensitivity of yeast *grx* mutants to external oxidants

Since it is not still possible to genetically manipulate AM fungi, functional analyses of the newly identified *R*. *irregularis GRX* genes were performed in a heterologous system. For this purpose, the *RiGRX3*, *RiGRX5* and *RiGRX6* full length cDNAs were cloned into the yeast expression vector pFL61 and tested for their ability to suppress the sensitivity of the *Δgrx3Δgrx4*, *Δgrx5* and *Δgrx6Δgrx7* disruption mutants of *S*. *cerevisiae* to external oxidants. In these experiments, *ScGrx3*, *ScGrx4* (functional homologs of *RiGRX4*), *ScGrx5* (homolog of *RiGRX5*) and *ScGrx6* (functional homolog of *RiGRX6*) were included as positive controls and the empty vector as negative control.

**Fig 3:**
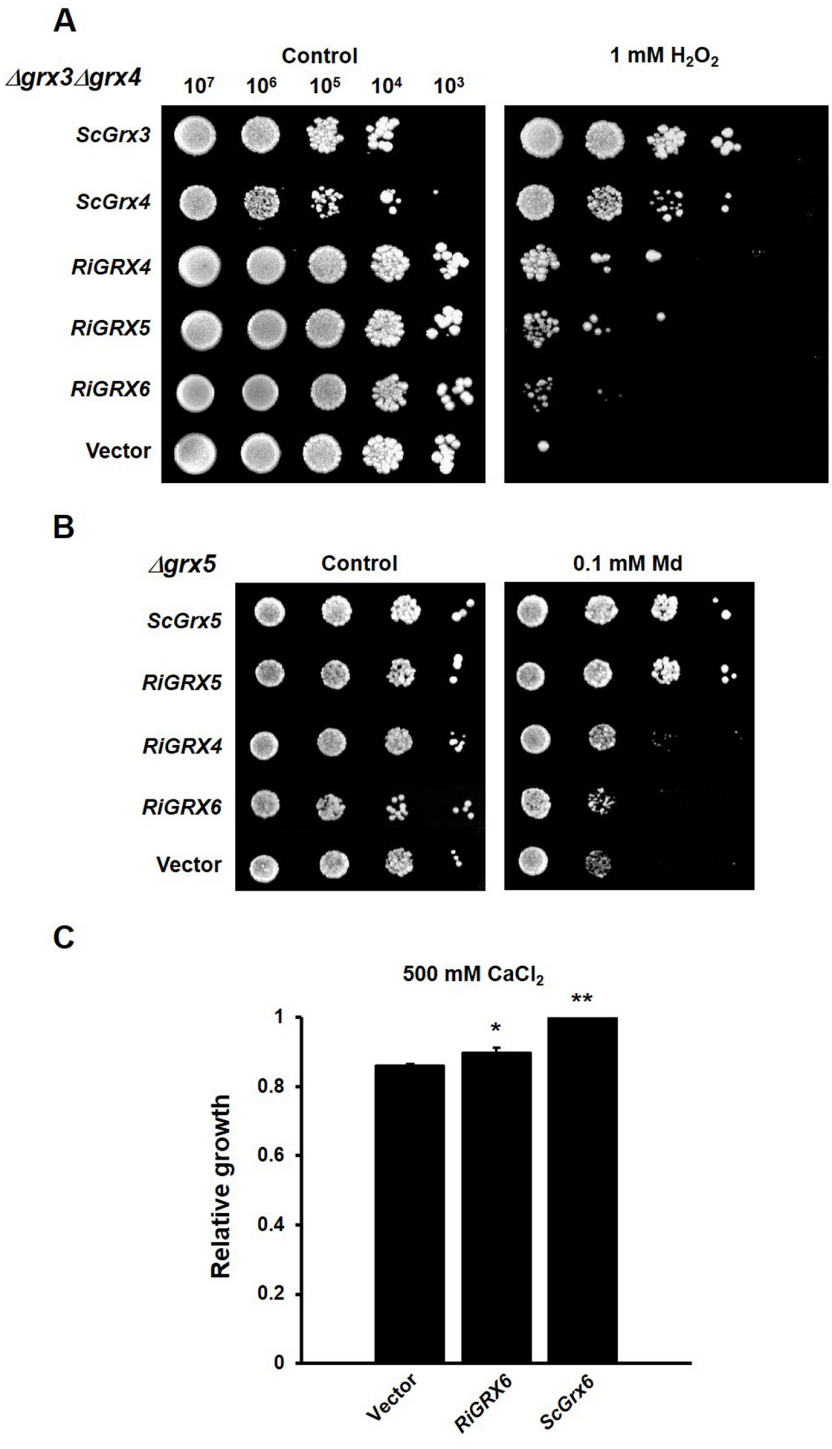
Complementation of the sensitivity to external oxidants of the *grx* yeast mutants by the *R*. *irregularis GRX* genes. **A.** Effect of *RiGRX4*, *RiGRX5* and *RiGRX6* expression on the sensitivity of the *Δgrx3Δgrx4* strain to 1 mM hydrogen peroxide (H_2_O_2_). **B.** Effect of *RiGRX4*, *RiGRX5* and *RiGRX6* expression on the sensitivity of the *Δgrx5* strain to 0.1 mM menadione (Md). The photographs were taken after 3 days of growth at 30°C. **C.** Effect of *RiGRX6* expression on the sensitivity of *Δgrx6Δgrx7* to 500 mM CaCl_2_ (40 h). Data are means of three independent experiments +/- standard error and represent the growth yield ratio between treated and untreated cultures and then made relative to this ratio in cells expressing the *S*. *cerevisiae Grx6*. Asterisks show statistically significant differences (p<0.05) relative to the strain transformed with the empty vector, according to the Fisher’s LSD test.

We first assessed whether RiGRX4 and RiGRX5, the two class II GRXs, could restore the inability of the *Δgrx3Δgrx4* double mutant to grow in the presence of hydrogen peroxide [43]. The *Δgrx3Δgrx4* yeast cells transformed with the empty vector did not grow on SD media supplemented with 1 mM hydrogen peroxide. Although less efficiently than yeast Grx3 and Grx4, expression of either *RiGRX4* or *RiGRX5* in the *Δgrx3Δgrx4* mutant yeast cells suppressed their sensitivity to hydrogen peroxide (Fig 3A). We also tested whether RiGRX4 and RiGRX5 could complement yeast Grx5 function and suppress the sensitivity of *Δgrx5* cells to menadione [42]. Neither the empty vector- nor the *RiGRX4*-transformed *Δgrx5* mutant cells were able to grow on media supplemented with menadione. However, the *RiGRX5*-expressing mutant yeast clearly grew in the presence of menadione (Fig 3B). Complementation of the two mutant strains by RiGRX5 suggests that in the heterologous system RiGRX5 is not only expressed in the mitochondria but also in the cytosol. From these data it is concluded that RiGRX4 and RiGRX5 play at least in the heterologous system an *in vivo* role in oxidative stress protection.

Capability of the *R*. *irregularis* GRX6 to revert sensitivity of the *Δgrx6Δgrx7* deletion mutant of *S*. *cerevisiae* to hydrogen peroxide was also assessed [44]. While expression of *ScGrx6* (positive control) in the *Δgrx6Δgrx7* mutant complemented its sensitivity to H_2_O_2_, RiGRX6 was not able to revert the mutant phenotype (data not shown). However, RiGRX6 was able to partially revert the hydrogen peroxide sensitivity of the *Δgrx3Δgrx4* mutant (Fig 3A), which suggests that the *RiGRX6* gene product has an antioxidant activity and that it should be located in the yeast cytosol.

Since the *S*. *cerevisiae* Grx6 has recently been shown to be involved in redox regulation of calcium homeostasis in yeast cells [58], we determined whether RiGRX6 could revert the increased sensitivity of the *Δgrx6Δgrx7* mutants to excess calcium. As shown in Fig 3C, the *RiGRX6* expressing cells were slightly less sensitive (29%) to excess calcium than the mutant cells transformed with the empty vector.

### Localization of *R*. *irregularis* GRXs in yeast

In an attempt to further understand the potential functions of RiGRX4, RiGRX5 and RiGRX6, localization of C-terminal GFP-tagged versions of these proteins in yeast was examined by fluorescence microscopy. For these purpose, the different *RiGRX-GFP* fusions were expressed in the *S*. *cerevisiae* deletion strains for their putative orthologues, except *RiGRX5-GFP* that was expressed in the *Δgrx3Δgrx4* strain due to the low viability of the *Δgrx5* transformants.

**Fig 4:**
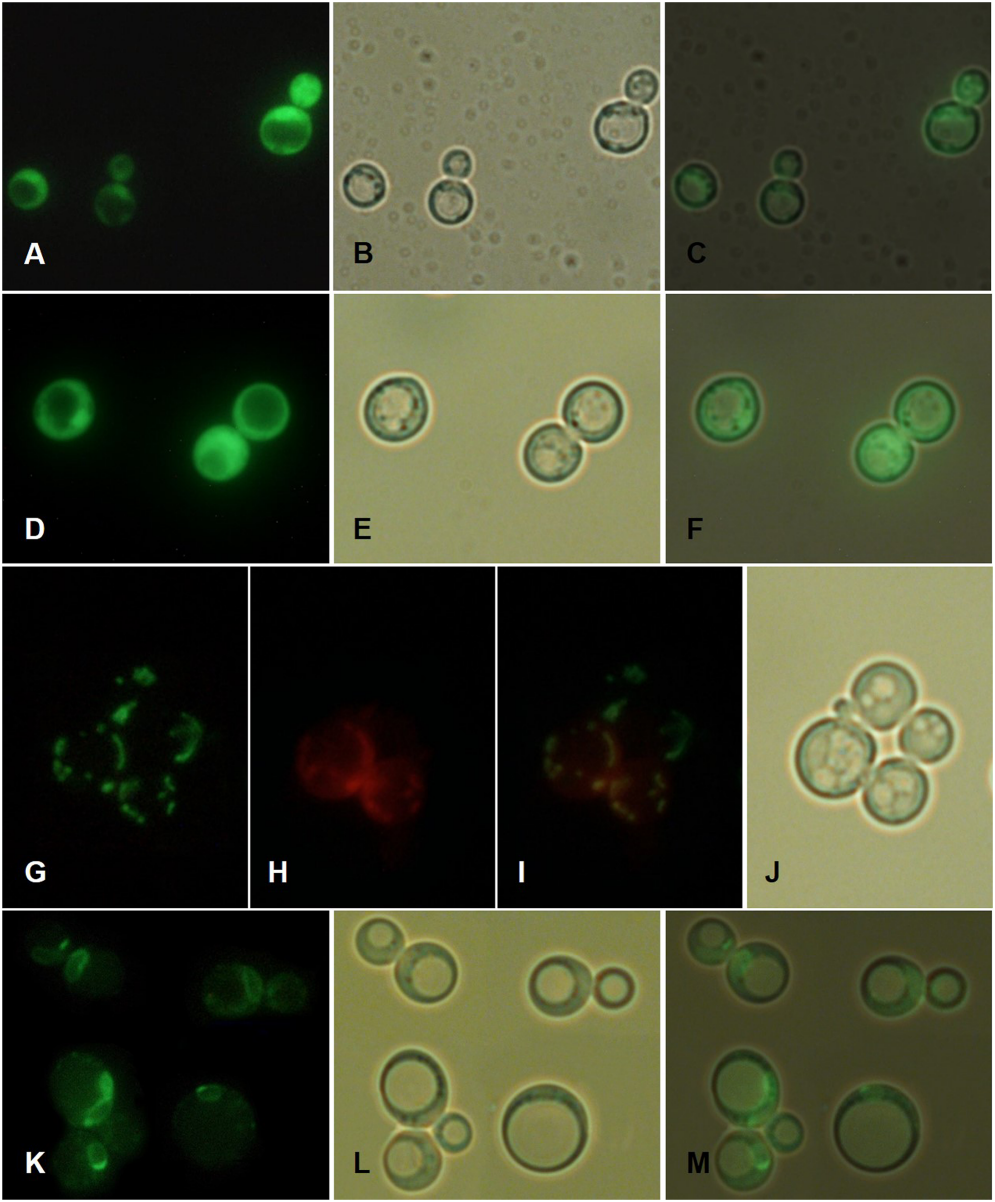
Localization of *R*. *irregularis* GRXs in *S*. *cerevisiae*. Soluble GFP (A-C) and C-terminal GFP-tagged versions of *RiGRX4* (D-F) and *RiGRX5* (G-J) were expressed in the *Δgrx3Δgrx4* cells. C-terminal GFP-tagged version of *RiGRX6* (K-M) was expressed in the *Δgrx6Δgrx7* yeast mutant cells. Cells were grown to mid-logarithmic phase and localization of fusion proteins was visualized by fluorescence microscopy (A, D, G and K). Mitochondria in the *RiGRX6*-GFP expressing cells were stained with MitoTracker^®^ Red and visualized by fluorescence microscopy (H). Bright field (B, E, J and L) and merged (C, F, I and M) images.

The yeast cells expressing RiGRX4-GFP fusion protein showed a general cytosolic fluorescence similar to the fluorescence of control cells expressing the soluble GFP (Fig 4A–4C), which indicates that RiGRX4 was targeted to the cytoplasm (Fig 4D–4F). RiGRX5-GFP was detected in the mitochondria, as determined by co-staining with the mitochondria-specific vital dye, MitoTracker^®^ Red (Fig 4G–4J), although a cytosolic signal was also observed in some cells (data not shown). The yeast cells expressing RiGRX6-GFP exhibited a perinuclear fluorescence pattern indicative of an ER localization [59], and an additional cytosolic fluorescence was observed in some cells (Fig 4K–4M). Detection of RiGRX5 and RiGRX6 in the yeast cytosol might be due to an overexpression artefact in the heterologous system.

### RiGRX4 and RiGRX5 play a role in Fe homeostasis in yeast

**Fig 5:**
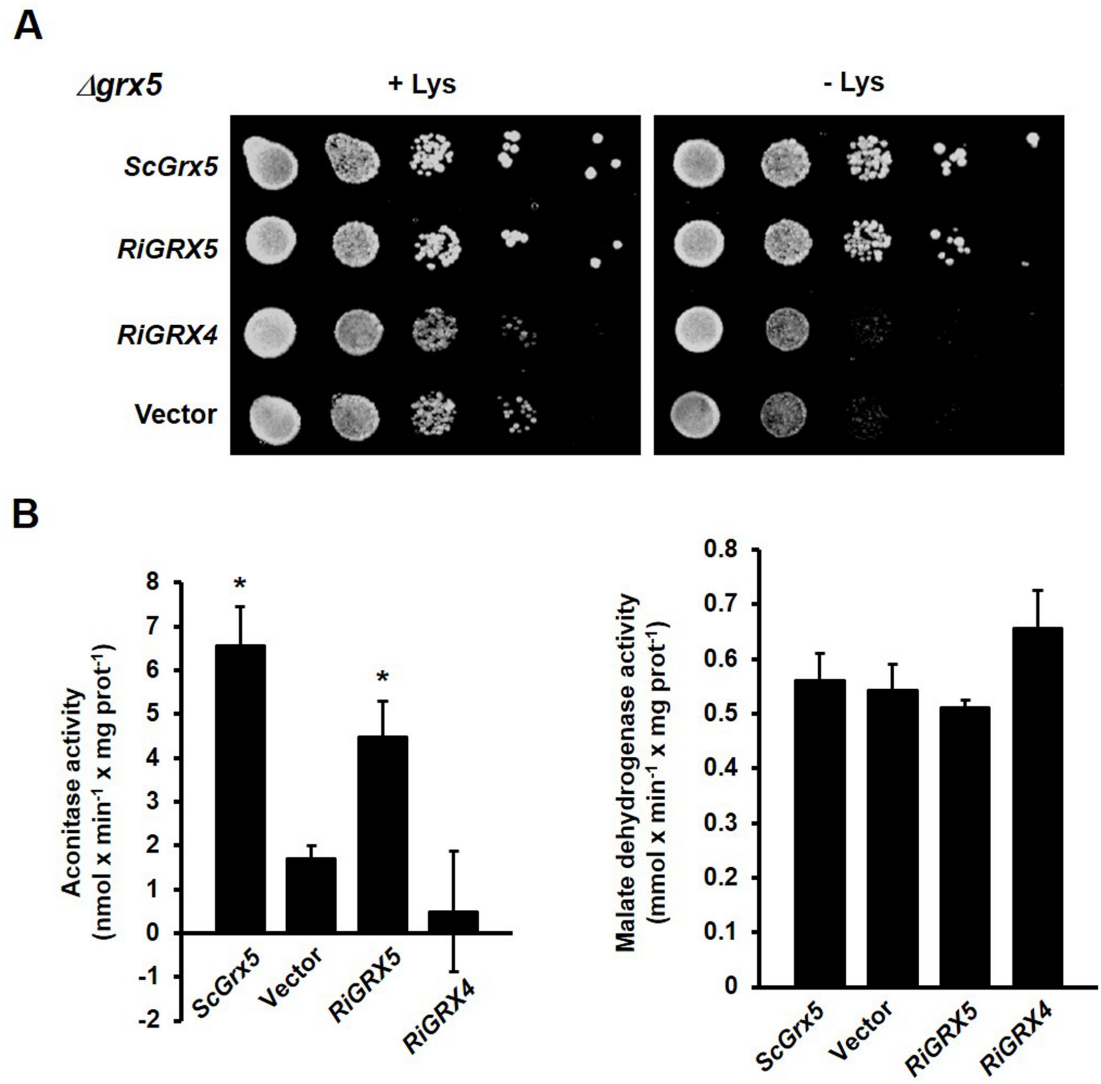
Analysis of the *in vivo* role of RiGRX5 in the biogenesis of Fe-S clusters in yeast. **A.** *Δgrx5* cells transformed with the empty vector or expressing *ScGrx5*, *RiGRX5* or *RiGRX4* were plated on SD medium with or without lysine. Plates were incubated at 30°C for 3 days. **B.** The activities of a Fe-S protein (aconitase) and a non-Fe-S protein (malate dehydrogenase) were determined in lysates of the *Δgrx5* cells transformed with the different constructs. Data are means +/- standard error. Asterisks show statistically significant differences (p<0.05) relative to the activities of the strain transformed with the empty vector, according to the Fisher’s LSD test.

Glutaredoxins with a CGFS domain in their active site have been shown to play a role in iron homeostasis. In *S*. *cerevisiae*, the three members of this subfamily participate in the synthesis of the iron-sulfur clusters in mitochondria (Grx5), or in signalling the iron status inside the cell for regulation of iron uptake and intracellular iron relocalization (Grx3 and Grx4). To investigate if the *R*. *irregularis* CGFS-type GRXs could play a role in iron homeostasis, we first determined the ability of RiGRX4 and RiGRX5, functional homologues of yeast Grx3/Grx4 and Grx5, respectively, to restore yeast Grx5 function. GRX5 deletion mutants fail to grow on lysine deficient media due to the inactivation of the mitochondrial Fe-S containing enzyme homoaconitase, an enzyme involved in lysine synthesis [30,42]. Expression of *RiGRX5*, but not of *RiGRX4*, clearly restored lysine auxotrophy of the *Δgrx5* cells to the same extent than the *S*. *cerevisiae Grx5* (Fig 5A). As expected, the aconitase activity was also restored in the *RiGRX5*-expressing yeast cells. However, the activity of the non Fe-S enzyme malate dehydrogenase was not affected by the expression of the *R*. *irregularis* CGFS-type GRXs (Fig 5B).

Deletion of *Grx5* and *Grx3*/*Grx4* in *S*. *cerevisiae* also results in an increase of the intracellular Fe levels. In the *Grx5* yeast mutants, increased iron levels are a consequence of the impairment of the biogenesis of Fe-S protein complexes in the absence of the GRX [42]. However, in the *Grx3*/*Grx4* mutants iron accumulation is the result of a constitutively activated cellular iron uptake system given that these GRXs play a central role in iron uptake regulation by interacting with the iron-responsive transcription factors Aft1 and Aft2, the major regulators of cellular iron uptake systems in yeast [60].

**Fig 6:**
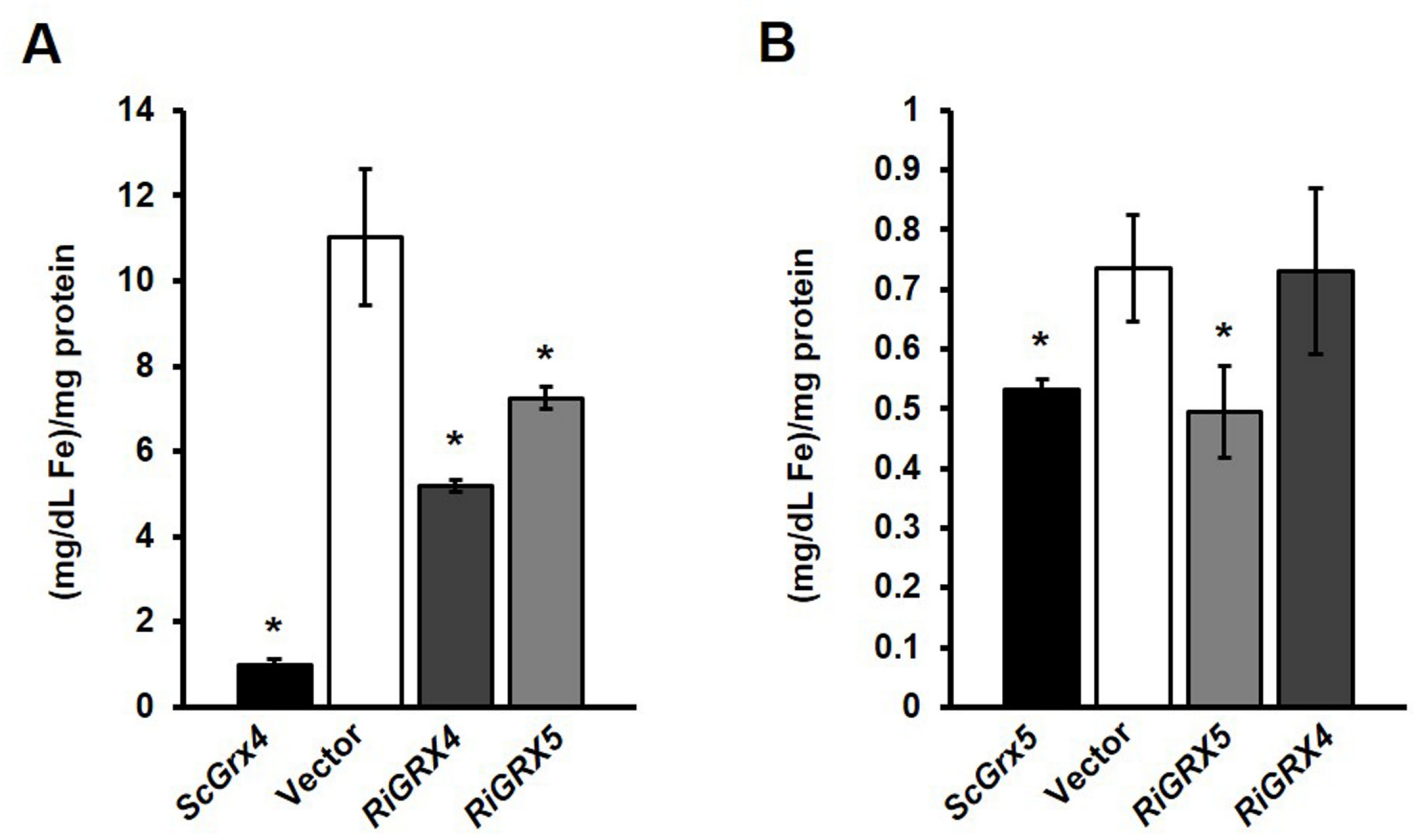
Analysis of the *in vivo* role of RiGRX4 and RiGRX5 in intracellular iron accumulation in *Δgrx3Δgrx4* (A) and *Δgrx5* (B) yeast mutant strains. Intracellular iron concentrations were determined in lysates of cells transformed with the empty vector or expressing *ScGrx4*, *ScGrx5*, *RiGRX4* or *RiGRX5*, with a Quantichron^™^ Iron Assay Kit. Data are means +/- standard error. Asterisks show statistically significant differences (p<0.1) relative to the values of the strain transformed with the empty vector (white columns), according to the Fisher’s LSD test.

To further understand the potential functions of RiGRX4 and RiGRX5, we assayed the intracellular iron content of the *Δgrx3Δgrx4* and *Δgrx5* yeast cells expressing *RiGRX4* or *RiGRX5*. Although less efficiently than *ScGrx4*, the gene products of *RiGRX4* and *RiGRX5* significantly suppressed intracellular iron accumulation of the *Δgrx3Δgrx4* yeast cells (Fig 6A). The decrease in iron accumulation was higher in the *RiGRX5*- (53% decrease) than in the *RiGRX4*-expressing (34% decrease) *Δgrx3Δgrx4* yeast cells. In the case of the *Δgrx5* strain, iron accumulation was suppressed in the mutant expressing *ScGrx5* and *RiGRX5* but not in the *RiGRX4*-expressing cells (Fig 6B). All these results indicate that both RiGRX4 and RiGRX5 could have an *in vivo* role on iron homeostasis in *R*. *irregularis*.

### Regulation of *RiGRXs* gene expression by hydrogen peroxide

**Fig 7:**
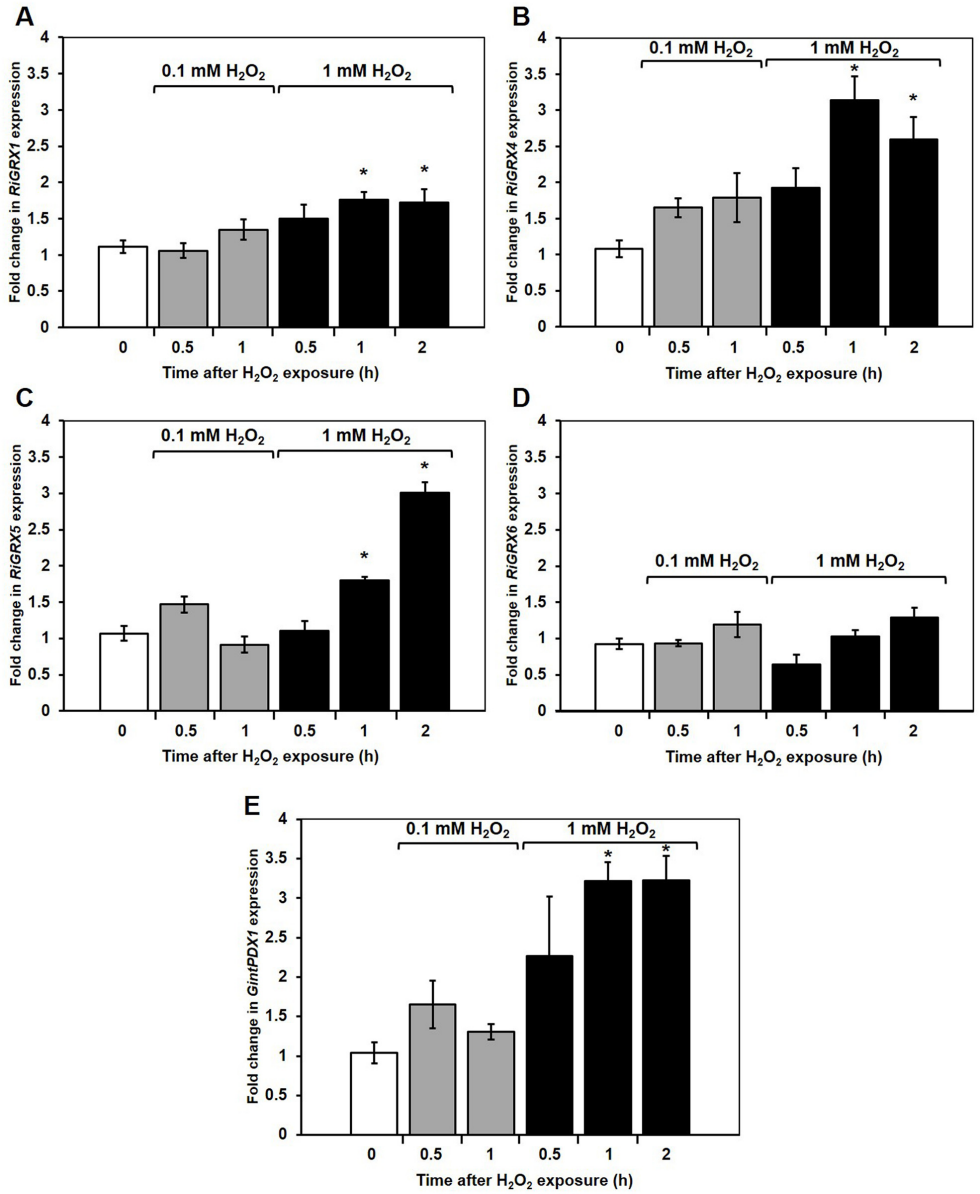
Effect of hydrogen peroxide on the expression of the *R*. *irregularis GRX* genes. *R*. *irregularis* ERM grown in M-C medium was exposed for different periods of time to 0.1 mM H_2_O_2_ (grey columns) or 1 mM H_2_O_2_ (black columns). *RiGRX1* (A), *RiGRX4* (B), *RiGRX5*(C), *RiGRX6* (D) and *GintPDX1* (E) gene expression. Data were normalized using the housekeeping gene *RiTEF*. Relative expression levels were calculated by the 2^-ΔΔCT^ method. Data are means +/- standard error. Asterisks show statistically significant differences (p<0.05) compared to the control value, according to the Fisher’s LSD test.

To investigate whether the RiGRXs could be involved in the response of *R*. *irregularis* to an oxidative stress, their gene expression was analyzed by real-time RT-PCR in the ERM that had been exposed for different periods of time to different concentrations of H_2_O_2_. Gene expression data are referred to the expression levels detected in mycelia from control plates. Expression of *GintPDX1*, a *R*. *irregularis* gene encoding a protein involved in vitamin B6 biosynthesis that is up-regulated by H_2_O_2_ [21], was also determined as a control of the H_2_O_2_ treatments. As expected, *GintPDX1* transcript levels increased 1 h after the addition of 1 mM H_2_O_2_ to the ERM. Up-regulation of *RiGRX1*, *RiGRX4* and *RiGRX5* gene expression was also observed 1 h after exposure of the fungus to 1 mM H_2_O_2_. However, *RiGRX6* gene expression was not significantly affected by the addition of H_2_O_2_ at any of the concentrations and time points analyzed (Fig 7).

### *RiGRX4* expression is up-regulated by iron

**Fig 8:**
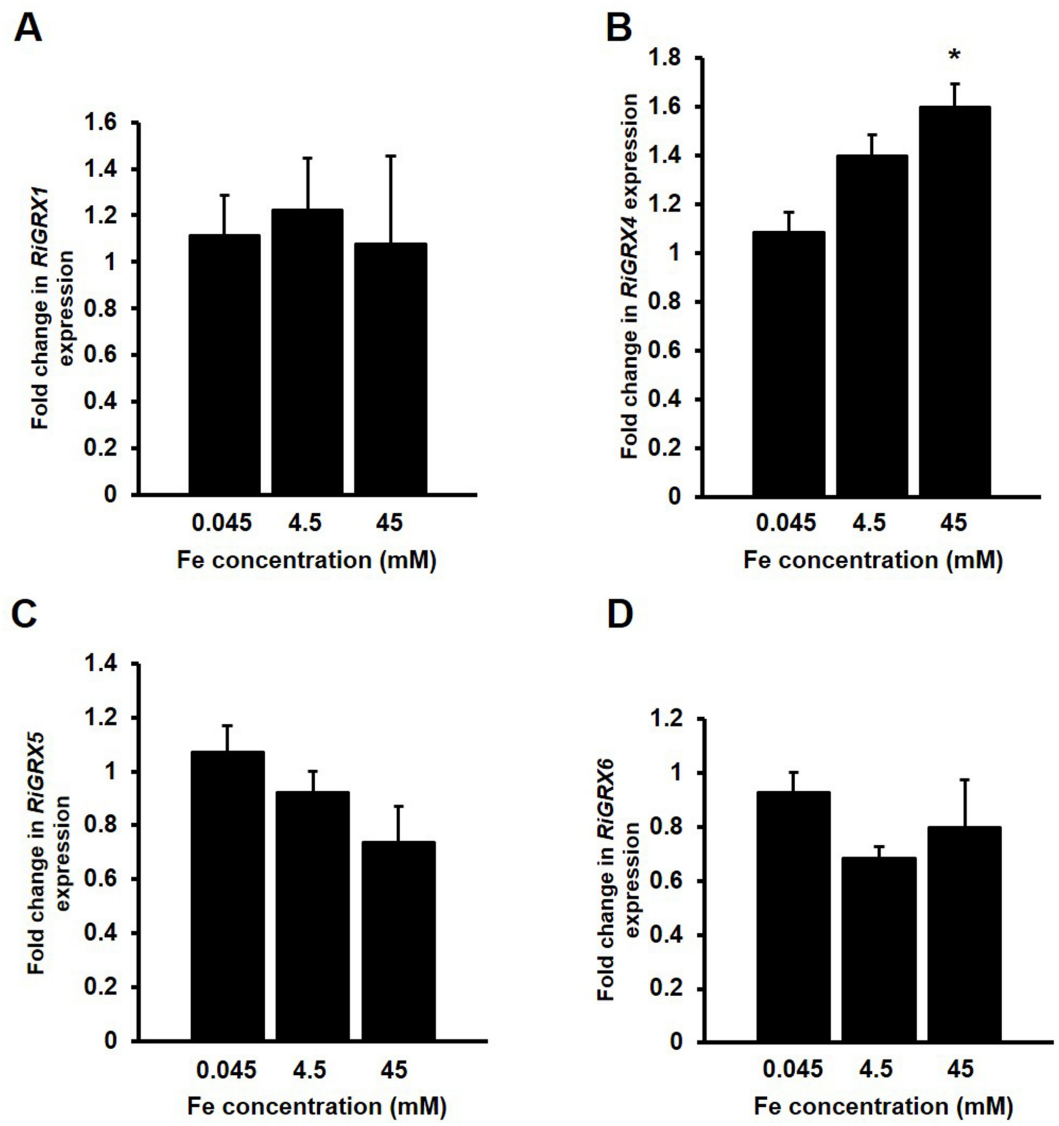
Effect of iron on the expression of the *R*. *irregularis GRX* genes. *R*. *irregularis* was grown in M-C media containing 45 μM Fe (control) or supplemented with 4.5 mM Fe or 45 mM Fe medium for 2 weeks. *RiGRX1* (A), *RiGRX4* (B), *RiGRX5* (C) and *RiGRX6* (D) gene expression. Data were normalized using the housekeeping gene *RiTEF*. Relative expression levels were calculated by the 2^-ΔΔCT^ method. Data are means +/- standard error. Asterisks show statistically significant differences (p<0.05) compared to the control value, according to the Fisher’s LSD test.

To assess if the *R*. *irregularis* GRXs could have an iron-related function, *RiGRXs* transcript levels were analysed in ERM grown in the presence of different iron concentrations (Fig 8). Relative to the ERM grown in M media containing 45 μM Fe, development of the fungus in the presence of 45 mM Fe induced a slight but statistically significant up-regulation of *RiGRX4* gene expression. Transcript levels of *RiGRX1*, *RiGRX5* and *RiGRX6* were not significantly affected by the amount of Fe present in the culture medium.

## Discussion

The GRX family comprises a large group of related enzymes that are ubiquitously present in different compartments of prokaryotic and eukaryotic cells. In this study, by using a wide-genome analysis of GRXs in the AM fungus *R*. *irregularis*, three new members of this family (RiGRX4, RiGRX5 and RiGRX6) were identified. Characterization of the newly identified genes showed that, in addition to the previously characterized cytosolic dithiol GRX [22], *R*. *irregularis* possesses three functional isoforms with a single cysteine residue in their active sites.

The *R*. *irregularis* GRXs are localized to different subcellular compartments. RiGRX1 and RiGRX4 were located in the cytosol, while RiGRX5 was targeted to the mitochondria and the cytosol, and RiGRX6 to the secretory pathway and the cytosol. Detection of RiGRX5 and RiGRX6 in the yeast cytosol explains why these proteins complemented the mutant phenotype of the *Δgrx3Δgrx4* strain, although expression of these proteins in the cytosol is most probably due to an artefact of the overexpression in the heterologous system. The *R*. *irregularis* GRX proteins were shown to suppress the mutant phenotypes of their yeast orthologs, suggesting that the biological function of this group of GRXs is evolutionary conserved. It is remarkable that RiGRX4, the *R*. *irregularis* GRX having a thioredoxin domain at the N-terminal end, possesses two GRX domains repeated in tandem, as it has been described for mammaliam and human GRX/PICOT proteins [61,62]. In contrast, in the rest of the reference fungi analyzed, except in *Rhizopus oryzeae*, these proteins contain only one GRX domain. This structure allows the binding of one or two Fe-S clusters which likely serve as redox sensors in response to redox signals for the GRX function in signal transduction and Fe-S cluster trafficking [63,64]. RiGRX6 clustered with the monothiol class I group but in a separate group to its yeast orthologs and similarly to the characterized *S*. *pombe* Grx3p has an extra N-terminal region with an unknown role [65]. While *S*. *cerevisiae* possesses two cytosolic class II GRXs (Grx3/4), *R*. *irregularis* has one (GRX4), similarly to *S*. *pombe* [33,66]. Class I GRXs come also in pairs in some organisms such as *S*. *cerevisiae*, having two CxxC GRXs (Grx1/2) and being the only fungus which has two GRXs belonging to the fungal-specific Grx6/Grx7 subfamily [26,62]. Nevertheless, our study showed that *R*. *irregularis* possesses only one homolog of each group of GRXs.

The finding that all the RiGRXs were able to supress sensitivity of the yeast GRX deletion mutants to external oxidants indicates that, at least in the heterologous system, these proteins play an *in vivo* role in oxidative stress protection. Up-regulation of *RiGRX1*, *RiGRX4* and *RiGRX5* gene expression in the *R*. *irregularis* ERM by H_2_O_2_ supports the hypothesis that these proteins are involved in the regulation of fungal redox processes. Oxidative stress broadly impact cells, initiating a series of redox-dependent modifications of proteins, lipids and nucleic acids. With respect to proteins, the thiol group of the cysteine residues is a major target of ROS. Reversible post-translational modifications of cysteine, such as glutathionylation, regulate protein activity and protect the thiol group during oxidative stress from irreversible oxidation. Although class II GRXs do not display *in vitro* deglutathionylation activity [26], the *S*. *cerevisiae* Grx5 was shown to deglutathionylate proteins *in vivo* [67]. Given that RiGRX1 was shown to perform a deglutathionylation reaction [22] and that function of the different types of GRXs is conserved through evolution [26], our data suggest a role for RiGRX1 and RiGRX5 in regulating the glutathionylation of thiols of cytosolic and mitochondrial target proteins, respectively, to protect the fungus from the oxidative stress induced by H_2_O_2_. However, given that multidomain GRXs do not seem to be involved in the reduction of oxidized proteins, that yeast Grx3 and Grx4 regulate actin cytoskeleton dynamics [68] and that actin is a known target for oxidation [69], it is tempting to hypothesize that RiGRX4 might be involved in the protection of the redox integrity of the actin cytoskeleton. On the other hand, the observation that RiGRX6 partially reverts the increased sensitivity of the *Δgrx6Δgrx7* mutants to excess calcium suggests that, similarly to ScGrx6, RiGRX6 might modulate intracellular calcium homeostasis in *R*. *irregularis*. However, further investigations are needed to confirm these hypotheses.

The ability of RiGRX4 and RiGRX5 to partially suppress iron accumulation in the *Δgrx3Δgrx4* mutant yeast and of RiGRX5 to restore the activity of the Fe-S containing protein aconitase and to suppress iron accumulation in *Δgrx5* yeast cells suggest that both proteins may be required for the biogenesis of Fe-S clusters and/or are involved in the regulation of iron homeostasis, as it has been shown for the majority of class II GRXs analysed to date [24]. Since sensitivity of the *Δgrx3Δgrx4* and *Δgrx5* cells to oxidizing agents is due to the iron-generated oxygen radicals via Fenton reaction [42,43] and since RiGRX4 and RiGRX5 reduced iron accumulation in the mutant yeasts, it is also possible that GRX4 and GRX5 protect the fungus from the oxidative damage induced by H_2_O_2_ by modulating iron homeostasis to control iron-generated oxygen radicals.

Gene expression data indicated that *RiGRX4* was the only *R*. *irregularis* GRX gene responsive to iron. In other fungi, class II or monothiol multidomain GRXs (Grx3/4) have been shown to be involved in iron uptake by interacting with iron-responsive transcription factors. In yeast under high-iron conditions, ScGrx3 and ScGrx4 interact with the transcription factors Aft1 and Aft2, excluding them from the nucleus and preventing the activation of several genes encoding proteins involved in iron uptake and distribution. Although no functional homologs of Aft1 and Aft2 have been found in the *R*. *irregularis* genome, an homologous sequence to the *S*. *pombe* iron-regulated transcription factor Fep1, which responds to high levels of iron as a negative regulator of the expression of several genes involved in iron acquisition [70], was found. These observations suggest that the mechanisms of transcriptional regulation of iron metabolism in *R*. *irregularis* might be more similar to those operating in *S*. *pombe* and ascomycete fungi than to *S*. *cerevisiae* and that up-regulation of *RiGRX4* gene expression under high Fe conditions might be involved in down-regulation of the high affinity Fe uptake systems.

*RiGRX4* and *RiGRX5* were found to be expressed at similar levels in the IRM and ERM. These data indicate that their encoded proteins might be required for maintaining the basal oxidative and iron metabolism of *R*. *irregularis*. The higher expression levels of *RiGRX1* and *RiGRX6* in the IRM suggests that their encoded proteins might play a role in the establishment of the symbiosis, as it has been shown for the class I or dithiol SmGRX1 and class II or monothiol SmGRX2 of *Sinorhizobium meliloti* [34]. Since RiGRX1 is a multifunctional protein that displays thiol oxidoreductase, peroxidase and glutathione-S-transferase activities [22], this protein should play a key role in oxidative stress protection of *R*. *irregularis* during its *in planta* phase, particularly through protein deglutathionylation. On the other hand, since fungal GRX6 type proteins have been proposed to be involved in redox regulation of calcium homeostasis [58], it is tempting to hypothesize that RiGRX6 activity might be involved in redox regulation of the *R*. *irregularis* calcium pumps and transporters that have been reported to be up-regulated during mycorrhiza development [71]. Unfortunately, the lack of standardized protocols for the genetic transformation in AM fungi precluded us from determining the precise *in vivo* roles of the *R*. *irregularis* GRXs.

In conclusion, this study shows that the AM fungus *R*. *irregularis* has four GRX members in its genome, and that the three monothiol GRXs identified might play a role in protection against ROS, as it was previously shown for RiGRX1. Furthermore, RiGRX4 and RiGRX5 might be involved in the regulation of iron metabolism. However, further analyses are necessary to determine the targets of RiGRXs and to fully understand the specific roles of the different GRX isoforms in *R*. *irregularis*.

## Supporting Information

S1 Fig Exon/intron organization of the *R*. *irregularis GRX* genes. Exon (E) and introns are represented by white and black boxes, respectively. The intron flanking sequences and the start and stop codons are indicated.
(TIF)

S1 Table Oligonucleotides used in this study. Overhangs are underlined (*Not*I (continuous lines) or *Sfi*I (dashed lines) restriction sites).
(PDF)
