# Supplementary figures and images for: Characterization of Three New Glutaredoxin Genes in the Arbuscular Mycorrhizal Fungus Rhizophagus irregularis: Putative Role of RiGRX4 and RiGRX5 in Iron Homeostasis
Source: PLoS One. 2016 Feb 22;11(2):e0149606. doi: 10.1371/journal.pone.0149606 (PMC4765768; doi:10.1371/journal.pone.0149606)

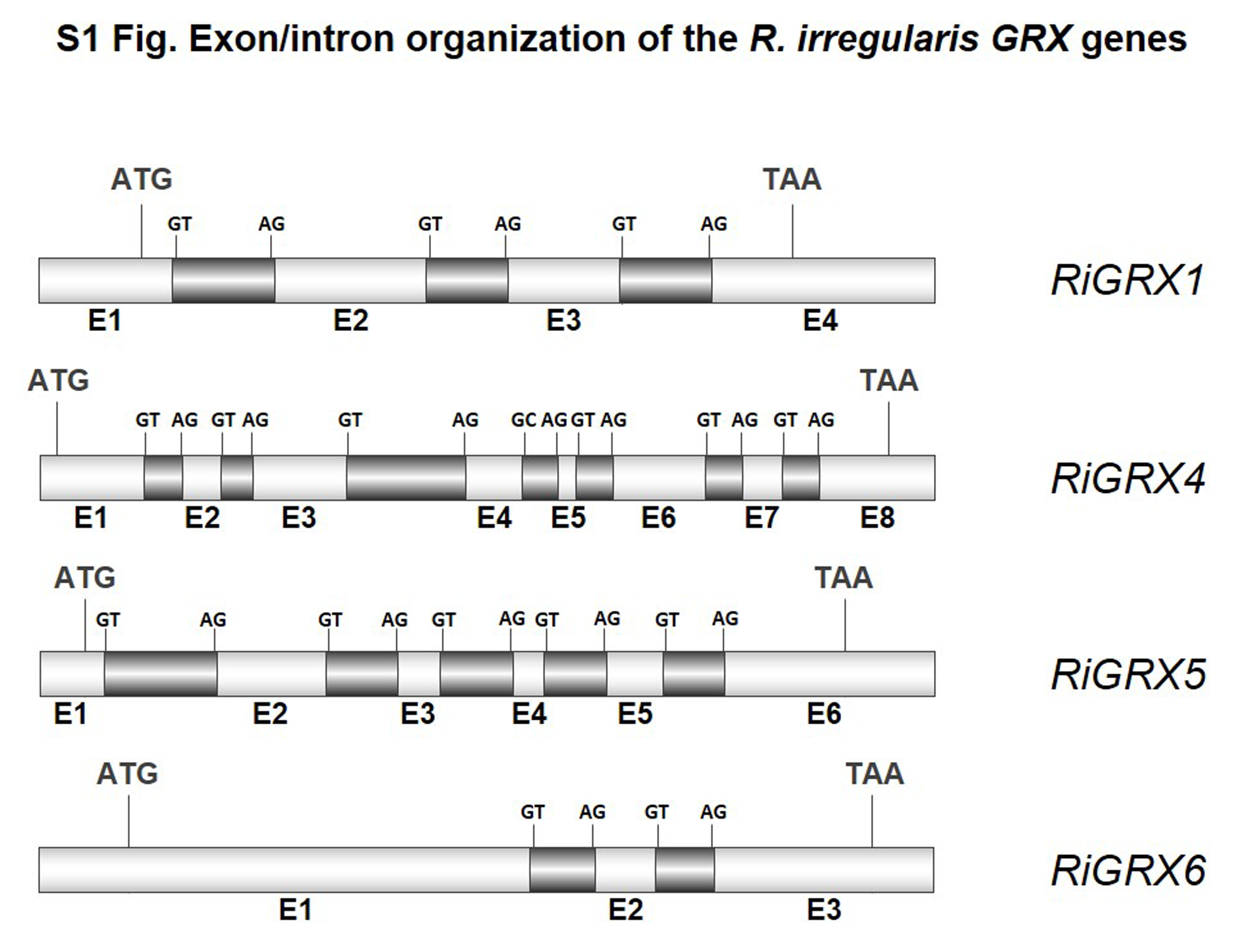

Supplement: S1 Fig — Exon (E) and introns are represented by white and black boxes, respectively. The intron flanking sequences and the start and stop codons are indicated. (TIF) [file pone.0149606.s001.tif]
