# Supplementary material for: Characterization of Three New Glutaredoxin Genes in the Arbuscular Mycorrhizal Fungus Rhizophagus irregularis: Putative Role of RiGRX4 and RiGRX5 in Iron Homeostasis
Source: PLoS One. 2016 Feb 22;11(2):e0149606. doi: 10.1371/journal.pone.0149606 (PMC4765768; doi:10.1371/journal.pone.0149606)
Supplement: S1 Table — Overhangs are underlined (NotI (continuous lines) or SfiI (dashed lines) restriction sites). (PDF) [file pone.0149606.s002.pdf]

S1 Table. Oligonucleotides used in this study

Primer	Sequence 5' - 3'
ScGRX3f	ATGTGTTCTTTTCAGGTTCCATCTG
ScGRX3r	TTAAGATTGGAGAGCATGCTGCAA
ScGRX4f	ATGACTGTGGTTGAAATAAAAAGC
ScGRX4r	TTACTGTAGAGCATGTTGGAAATA
ScGRX5f	ATGTTTCTCCCAAAATTCAATCC
ScGRX5r	TCAACGATCTTTGGTTTCTTCTTC
Grx4.fF	GCGGCCGCATGGACAGCAACTTG
Grx4.fR	GCGGCCGCTCAAATTGATTTCTCTAA
Grx5.fF	GCGGCCGCATGAGCTTACGATCATTA
Grx5.fR	GCGGCCGCTTAATTTTCCTCCTGTTC
Grx7.fF	ATGTCTAAAGCGGCGATCTTACC
Grx7.fR	TTATAAAGATGGTCGAAAAATTTCGTG
GintGRXfw2	GAAGATTCCGAAGGAAGAGC
GintGRXrev2	CAACGTGTTGACCCTTGATA
RiGrx4.qF	GATTTTTGGGCAACATGGGC
RiGrx4.qR	CGGGGAATTTCTCAGCCTCAATCT
RiGrx5.qF	GCGCAAGGTTTGGATTTCT
RiGrx5.qR	AGGGACTGTTGGCCATTGT
RiGrx7.qF	TGGGAGAAGGAGGTGAAGAA
RiGrx7.qR	CCAATGCTTCCTCGAAACG
GintPDXfw	CTGGAGATCCTGCTAAAAGAGC
GintPDXrev	CCAAGATCCTCCGATACTTCG
GintEFfw	GCTATTTTGATCATTGCCGCC
GintEFrev	TCATTAAAACGTTCTTCCGACC
RiGRX4-sfi-F	CAAGGCCATTACGGCCATGGACAGCAACTTGATAGAACTTAC
RiGRX4-sfi-R	GATGGCCGAGGCGGCCGAAATTGATTTCTCTAAAGCAAACATGG
RiGRX5-sfi-F	CAAGGCCATTACGGCCATGAGCTTACGATCATTAACACGTC
RiGRX5-sfi-R	GATGGCCGAGGCGGCCGATTTTTCCTCCTGTTCTAATTCATTTTTAG
RiGRX6-sfi-F	CAAGGCCATTACGGCCATGTCTAAAGCGGCGATCTTAC
RiGRX6-sfi-R	GATGGCCGAGGCGGCCGATAAAGATGGTCGAAAAATTTCGTG
